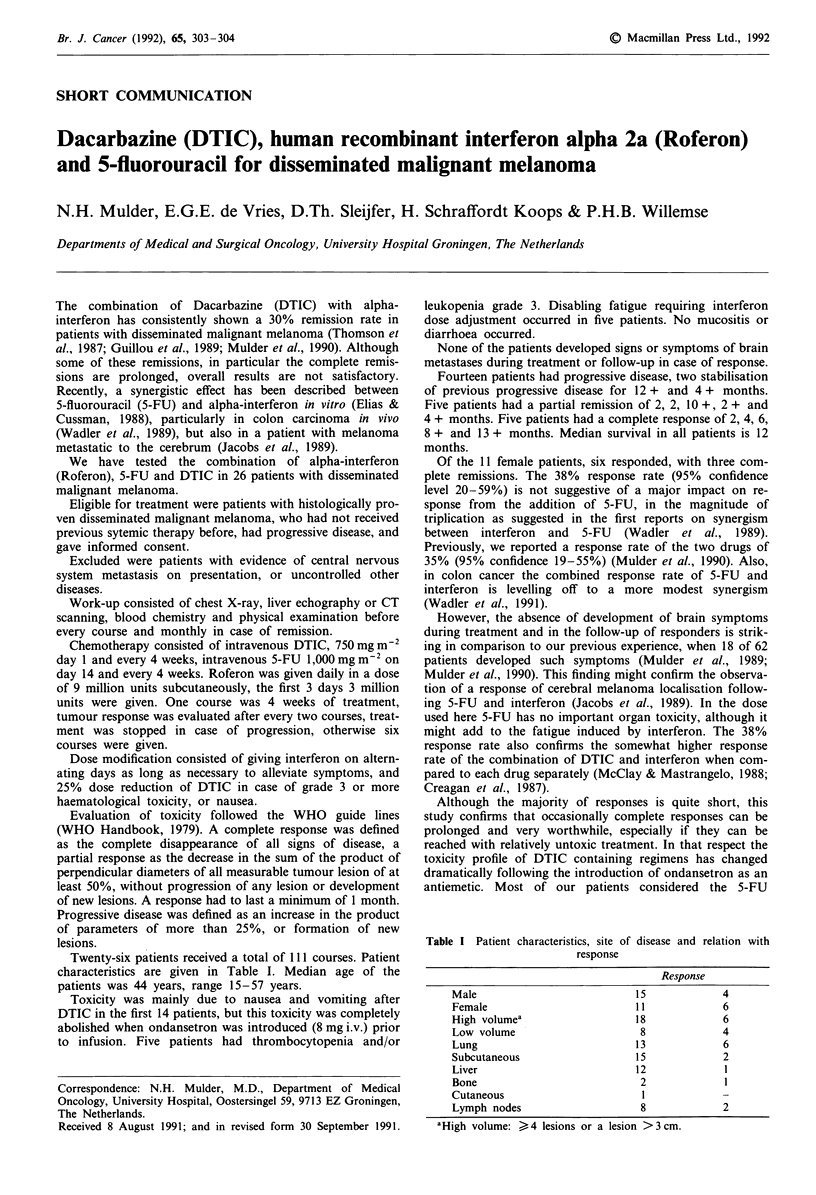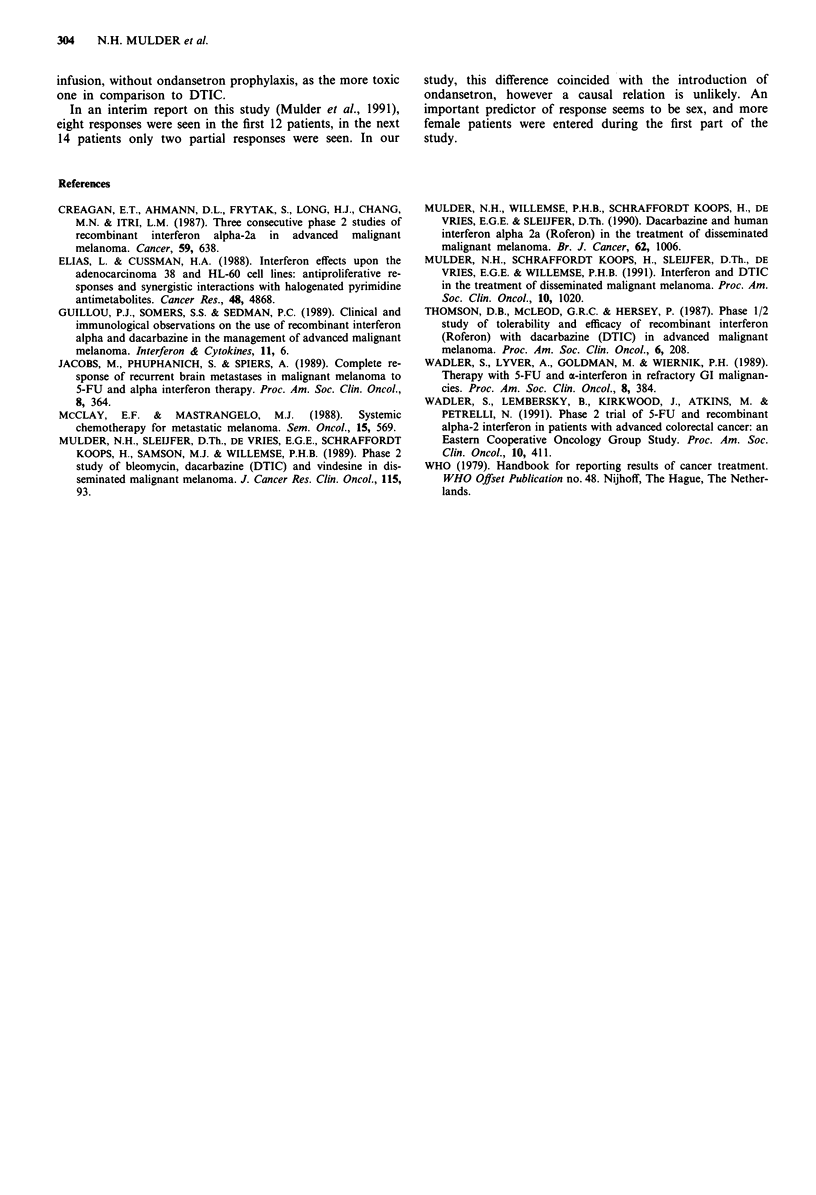# Dacarbazine (DTIC), human recombinant interferon alpha 2a (Roferon) and 5-fluorouracil for disseminated malignant melanoma.

**DOI:** 10.1038/bjc.1992.61

**Published:** 1992-02

**Authors:** N. H. Mulder, E. G. de Vries, D. T. Sleijfer, H. Schraffordt Koops, P. H. Willemse

**Affiliations:** Department of Medical Oncology, University Hospital Groningen, The Netherlands.


Br. J. Cancer (1992), 65, 303-304 © Macmillan Press Ltd., 1992

SHORT COMMUNICATION

### Dacarbazine (DTIC), human recombinant interferon alpha 2a (Roferon) and 5-fluorouracil for disseminated malignant melanoma

N.H. Mulder, E.G.E. de Vries, D.Th. Sleijfer, H. Schraffordt Koops & P.H.B. Willemse

*Departments of Medical and Surgical Oncology, University Hospital Groningen, The Netherlands*

The combination of Dacarbazine (DTIC) with alpha-interferon has consistently shown a 30% remission rate in patients with disseminated malignant melanoma (Thomson *et al.*, 1987; Guillou *et al.*, 1989; Mulder *et al.*, 1990). Although some of these remissions, in particular the complete remissions are prolonged, overall results are not satisfactory. Recently, a synergistic effect has been described between 5-fluorouracil (5-FU) and alpha-interferon *in vitro* (Elias & Cussman, 1988), particularly in colon carcinoma *in vivo* (Wadler *et al.*, 1989), but also in a patient with melanoma metastatic to the cerebrum (Jacobs *et al.*, 1989).

We have tested the combination of alpha-interferon (Roferon), 5-FU and DTIC in 26 patients with disseminated malignant melanoma.

Eligible for treatment were patients with histologically proven disseminated malignant melanoma, who had not received previous sytemic therapy before, had progressive disease, and gave informed consent.

Excluded were patients with evidence of central nervous system metastasis on presentation, or uncontrolled other diseases.

Work-up consisted of chest X-ray, liver echography or CT scanning, blood chemistry and physical examination before every course and monthly in case of remission.

Chemotherapy consisted of intravenous DTIC, 750 mg $m^{-2}$ day 1 and every 4 weeks, intravenous 5-FU 1,000 mg $m^{-2}$ on day 14 and every 4 weeks. Roferon was given daily in a dose of 9 million units subcutaneously, the first 3 days 3 million units were given. One course was 4 weeks of treatment, tumour response was evaluated after every two courses, treatment was stopped in case of progression, otherwise six courses were given.

Dose modification consisted of giving interferon on alternating days as long as necessary to alleviate symptoms, and 25% dose reduction of DTIC in case of grade 3 or more haematological toxicity, or nausea.

Evaluation of toxicity followed the WHO guide lines (WHO Handbook, 1979). A complete response was defined as the complete disappearance of all signs of disease, a partial response as the decrease in the sum of the product of perpendicular diameters of all measurable tumour lesion of at least 50%, without progression of any lesion or development of new lesions. A response had to last a minimum of 1 month. Progressive disease was defined as an increase in the product of parameters of more than 25%, or formation of new lesions.

Twenty-six patients received a total of 111 courses. Patient characteristics are given in Table I. Median age of the patients was 44 years, range 15–57 years.

Toxicity was mainly due to nausea and vomiting after DTIC in the first 14 patients, but this toxicity was completely abolished when ondansetron was introduced (8 mg i.v.) prior to infusion. Five patients had thrombocytopenia and/or leukopenia grade 3. Disabling fatigue requiring interferon dose adjustment occurred in five patients. No mucositis or diarrhoea occurred.

None of the patients developed signs or symptoms of brain metastases during treatment or follow-up in case of response.

Fourteen patients had progressive disease, two stabilisation of previous progressive disease for 12+ and 4+ months. Five patients had a partial remission of 2, 2, 10+, 2+ and 4+ months. Five patients had a complete response of 2, 4, 6, 8+ and 13+ months. Median survival in all patients is 12 months.

Of the 11 female patients, six responded, with three complete remissions. The 38% response rate (95% confidence level 20–59%) is not suggestive of a major impact on response from the addition of 5-FU, in the magnitude of triplication as suggested in the first reports on synergism between interferon and 5-FU (Wadler *et al.*, 1989). Previously, we reported a response rate of the two drugs of 35% (95% confidence 19–55%) (Mulder *et al.*, 1990). Also, in colon cancer the combined response rate of 5-FU and interferon is levelling off to a more modest synergism (Wadler *et al.*, 1991).

However, the absence of development of brain symptoms during treatment and in the follow-up of responders is striking in comparison to our previous experience, when 18 of 62 patients developed such symptoms (Mulder *et al.*, 1989; Mulder *et al.*, 1990). This finding might confirm the observation of a response of cerebral melanoma localisation following 5-FU and interferon (Jacobs *et al.*, 1989). In the dose used here 5-FU has no important organ toxicity, although it might add to the fatigue induced by interferon. The 38% response rate also confirms the somewhat higher response rate of the combination of DTIC and interferon when compared to each drug separately (McClay & Mastrangelo, 1988; Creagan *et al.*, 1987).

Although the majority of responses is quite short, this study confirms that occasionally complete responses can be prolonged and very worthwhile, especially if they can be reached with relatively untoxic treatment. In that respect the toxicity profile of DTIC containing regimens has changed dramatically following the introduction of ondansetron as an antiemetic. Most of our patients considered the 5-FU

**Table I** Patient characteristics, site of disease and relation with response

| | | *Response* |
|---|---|---|
| Male | 15 | 4 |
| Female | 11 | 6 |
| High volume[a] | 18 | 6 |
| Low volume | 8 | 4 |
| Lung | 13 | 6 |
| Subcutaneous | 15 | 2 |
| Liver | 12 | 1 |
| Bone | 2 | 1 |
| Cutaneous | 1 | – |
| Lymph nodes | 8 | 2 |

[a]High volume: ⩾4 lesions or a lesion >3 cm.

Correspondence: N.H. Mulder, M.D., Department of Medical Oncology, University Hospital, Oostersingel 59, 9713 EZ Groningen, The Netherlands.
Received 8 August 1991; and in revised form 30 September 1991.

infusion, without ondansetron prophylaxis, as the more toxic one in comparison to DTIC.

In an interim report on this study (Mulder *et al.*, 1991), eight responses were seen in the first 12 patients, in the next 14 patients only two partial responses were seen. In our study, this difference coincided with the introduction of ondansetron, however a causal relation is unlikely. An important predictor of response seems to be sex, and more female patients were entered during the first part of the study.

### References

CREAGAN, E.T., AHMANN, D.L., FRYTAK, S., LONG, H.J., CHANG, M.N. & ITRI, L.M. (1987). Three consecutive phase 2 studies of recombinant interferon alpha-2a in advanced malignant melanoma. *Cancer*, **59**, 638.

ELIAS, L. & CUSSMAN, H.A. (1988). Interferon effects upon the adenocarcinoma 38 and HL-60 cell lines: antiproliferative responses and synergistic interactions with halogenated pyrimidine antimetabolites. *Cancer Res.*, **48**, 4868.

GUILLOU, P.J., SOMERS, S.S. & SEDMAN, P.C. (1989). Clinical and immunological observations on the use of recombinant interferon alpha and dacarbazine in the management of advanced malignant melanoma. *Interferon & Cytokines*, **11**, 6.

JACOBS, M., PHUPHANICH, S. & SPIERS, A. (1989). Complete response of recurrent brain metastases in malignant melanoma to 5-FU and alpha interferon therapy. *Proc. Am. Soc. Clin. Oncol.*, **8**, 364.

MCCLAY, E.F. & MASTRANGELO, M.J. (1988). Systemic chemotherapy for metastatic melanoma. *Sem. Oncol.*, **15**, 569.

MULDER, N.H., SLEIJFER, D.Th., DE VRIES, E.G.E., SCHRAFFORDT KOOPS, H., SAMSON, M.J. & WILLEMSE, P.H.B. (1989). Phase 2 study of bleomycin, dacarbazine (DTIC) and vindesine in disseminated malignant melanoma. *J. Cancer Res. Clin. Oncol.*, **115**, 93.

MULDER, N.H., WILLEMSE, P.H.B., SCHRAFFORDT KOOPS, H., DE VRIES, E.G.E. & SLEIJFER, D.Th. (1990). Dacarbazine and human interferon alpha 2a (Roferon) in the treatment of disseminated malignant melanoma. *Br. J. Cancer*, **62**, 1006.

MULDER, N.H., SCHRAFFORDT KOOPS, H., SLEIJFER, D.Th., DE VRIES, E.G.E. & WILLEMSE, P.H.B. (1991). Interferon and DTIC in the treatment of disseminated malignant melanoma. *Proc. Am. Soc. Clin. Oncol.*, **10**, 1020.

THOMSON, D.B., MCLEOD, G.R.C. & HERSEY, P. (1987). Phase 1/2 study of tolerability and efficacy of recombinant interferon (Roferon) with dacarbazine (DTIC) in advanced malignant melanoma. *Proc. Am. Soc. Clin. Oncol.*, **6**, 208.

WADLER, S., LYVER, A., GOLDMAN, M. & WIERNIK, P.H. (1989). Therapy with 5-FU and α-interferon in refractory GI malignancies. *Proc. Am. Soc. Clin. Oncol.*, **8**, 384.

WADLER, S., LEMBERSKY, B., KIRKWOOD, J., ATKINS, M. & PETRELLI, N. (1991). Phase 2 trial of 5-FU and recombinant alpha-2 interferon in patients with advanced colorectal cancer: an Eastern Cooperative Oncology Group Study. *Proc. Am. Soc. Clin. Oncol.*, **10**, 411.

WHO (1979). Handbook for reporting results of cancer treatment. *WHO Offset Publication* no. 48. Nijhoff, The Hague, The Netherlands.